# The Function of Anal Fin Egg-Spots in the Cichlid Fish *Astatotilapia burtoni*

**DOI:** 10.1371/journal.pone.0029878

**Published:** 2012-01-05

**Authors:** Anya Theis, Walter Salzburger, Bernd Egger

**Affiliations:** Zoological Institute, University of Basel, Basel, Switzerland; Biodiversity Insitute of Ontario - University of Guelph, Canada

## Abstract

Color and pigmentation patterns of animals are often targets of sexual selection because of their role in communication. Although conspicuous male traits are typically implicated with intersexual selection, there are examples where sex-specific displays play a role in an intrasexual context, e.g. when they serve as signals for aggression level and/or status. Here, we focus on the function of a conspicuous male ornament in the most species-rich tribe of cichlid fishes, the haplochromines. A characteristic feature of these ca. 1500 species are so-called egg-spots in form of ovoid markings on the anal fins of males, which are made up of carotenoid based pigment cells. It has long been assumed that these yellow, orange or reddish egg-spots play an important role in the courtship and spawning behavior of these maternal mouth-brooding fishes by mimicking the eggs of a conspecific female. The exact function of egg-spots remains unknown, however, and there are several hypotheses about their mode of action. To uncover the function of this cichlid-specific male ornament, we used female mate choice experiments and a male aggression test in the haplochromine species *Astatotilapia burtoni*. We manipulated the number and arrangement of egg-spots on the anal fins of males, or removed them entirely, and tested (*1*) female preference with visual contact only using egg-traps, (*2*) female preference with free contact using paternity testing with microsatellites and (*3*) male aggression. We found that females did not prefer males with many egg-spots over males with fewer egg-spots and that females tended to prefer males without egg-spots over males with egg-spots. Importantly, males without egg-spots sired clutches with the same fertilization rate as males with egg-spots. In male aggression trials, however, males with fewer egg-spots received significantly more attacks, suggesting that egg-spots are an important signal in intrasexual communication.

## Introduction

Since the publication of Charles R. Darwin's second most famous book ‘The Descent of Man’ in 1871 [1], sexual selection has been recognized as being important for speciation because it can mediate reproductive isolation [2], [3]. Darwin differentiated between two fundamental modes of sexual selection: (*i*) competition between members of the same sex (often males) for reproductive opportunity (‘intrasexual’), and (*ii*) active mate choice of members from one sex (often females) for certain members from the other sex (‘intersexual’). Particularly in the latter mode, mate choice is often based on visual ornaments, although color traits can serve with respect to both inter- and intrasexual communication and, hence, inter- and intrasexual selection [4], [5]. Moreover, there are instances where the role of ornaments was altered from a function in female choice to one in male-male competition or *vice versa* [6].

Color and pigmentation patterns seem to play a central role in the explosively radiating cichlid fish species in the East African Great Lakes in general, and in the haplochromine cichlids in particular [7], [8], [9], [10]. Haplochromines contain the vast majority of East African cichlid species with the entire species flocks of lakes Victoria (ca. 700 species) and Malawi (ca. 700 species), the tribe Tropheini from Lake Tanganyika (ca. 25 species) and most riverine East African cichlids (ca. 200 species) (see e.g. [11], [12]). Therefore haplochromines are not only the – by far – most species-rich tribe of cichlid fishes but also a model of radiating species. A prominent feature of the haplochromines is their wealth of color morphs and their sexual color dimorphism, which is what led many authors to postulate an important evolutionary role of sexual selection via female mate choice [13], [14], [15], [16]. Interestingly, all haplochromines are maternal mouthbrooders where females incubate the eggs in their buccal cavities (see e.g. [12], [17]). Mouthbrooding evolved from substrate spawning several times during cichlid evolution [18], but only the ‘modern haplochromines’ show a derived polygynous or polygynandrous maternal mouthbrooding system with males carrying egg-spots on their anal fins [12], [17], [19]. These ovoid markings consist of a transparent outer ring and a brightly colored yellow, orange or reddish center [17], [20], [21]. The conspicuous central area is formed by xanthophores – a pigment cell type containing carotenoids and pteridines [19], [22].

**Figure 1:**
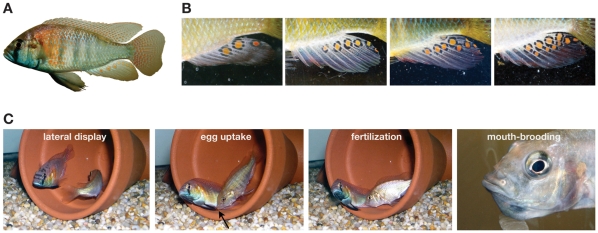
The egg-spots of haplochromine cichlids as exemplified in *Astatotilapia burtoni*. (A) A male of *A. burtoni* showing egg-spots on its anal fin. (B) Natural variation of egg-spots in *A. burtoni*. All these fish were caught and photographed at the south-eastern part of Lake Tanganyika in Zambia. (C) A typical courtship and mating cycle of a haplochromine starts with a lateral display of the male, to which the female responds; she then lays a clutch of eggs and immediately takes them up into her mouth. The male then presents the egg-spots on the anal fin; the female seemingly nuzzles at these egg-spots and the male releases sperm so that the eggs are fertilized within the females' mouth. The eggs and larvae then stay in the buccal cavity of the female for a period of several days to a few weeks. The arrow points to the location of an egg that the female is taking up into her mouth.

Egg-spots appear to be important in the courtship and spawning behavior of haplochromines [20], [21], [23], [24] (Figure 1C). The exact function of egg-spots is unknown, however, and several hypotheses exist that seek to explain their mode of action and their evolutionary origin.

Wickler [20], [21] suggested that egg-spots on the male's anal fin mimic real eggs of a species and therefore function as signal (‘releaser’) during courtship and to maximize fertilization rates. The egg mimicry hypothesis is primarily based on the putatively similar appearance in shape and coloration of egg-spots and the eggs of the respective species [20], [21], [25]. However, egg-spots and eggs often do not match in size, shape and coloration, which is inconsistent with the mimicry hypothesis [26], [27]. Still, this mismatch between real and ‘dummy’ eggs may be due to a trade-off between attractiveness towards females and conspicuousness for predators [24]. Hert [23] was the first to experimentally test the egg-dummy scenario. She showed that in the species *Astatotilapia elegans* there were no differences in fertilization rates between males without and males with intact egg-spots, which at least partly contradicted the mimicry hypothesis. On the other hand, males with intact egg-spots fertilized twice as many clutches compared to males without egg-spots. Further mate choice trials revealed that females always chose males with egg-spots and preferred males with four egg-spots over males with one egg-spot. In *Pseudotropheus aurora* (now *Maylandia aurora*), females spawned more frequently with males displaying more egg-spots and male egg-spot number correlated significantly with the number of fertilized clutches [28]. Hert [23], [28] concluded that egg-spots serve as sexual advertisement and that disruptive selection on male egg-spots may have contributed to reproductive isolation and, hence, speciation. In mate choice trials with *Pseudotropheus lombardoi* (now *Maylandia lombardoi*), a Lake Malawi cichlid in which males display a single egg-spot, females preferred males with one egg-spot over males with an artificially added second one [29]. Couldridge [29] suggested that female preference maintains the single egg-spot in *P. lombardoi* and that egg-spots may be linked to species recognition.

Previous hypotheses regarding the function of egg-spots involve female choice as the main explanation for the maintenance of this conspicuous male trait (see above). Interestingly, however, essential sequences of courtship behavior like quivering and lateral display are also used in male-male interactions. When males fight, which happens frequently in territorial haplochromines, they quiver, move back and forward and attack sideways [30]. So, why shouldn't egg-spots play a role in male-male competition, too? There are several arguments that would implicate egg-spots with intrasexual selection. Importantly, egg-spots are, most likely, an honest signal of male quality, as carotenoids cannot be synthesized *de novo* by animals (pteridines, on the other hand, can be synthesized; yet, this process appears to also be costly) [31], [32]. There is evidence that dominant males often display more egg-spots [33], [34]. Moreover, competition among males appears as yet another important component of color evolution in cichlids [35], [36].

To understand the function of egg-spots in the haplochromine cichlid *Astatotilapia burtoni*, we conducted three experiments. First, females had a choice based on visual cues only between two size-matched males differing in egg-spot number (one trial with naturally varying numbers of egg-spots (experiment 1.1) and one trial with manipulated numbers of egg-spots (experiment 1.2)). Second, we performed a female four-way choice experiment in a partial partition set-up (see e.g. [16]), in which females had the choice between four size-matched males with manipulated egg-spot numbers; we measured fertilization rate and genotyped the offspring in order to assess female preference by determining paternity (experiment 2). Finally, we conducted male aggression trials to test for a potential role of egg-spots in male-male competition (experiment 3).

## Results

### Experiment 1: female two-way choice

**Figure 2:**
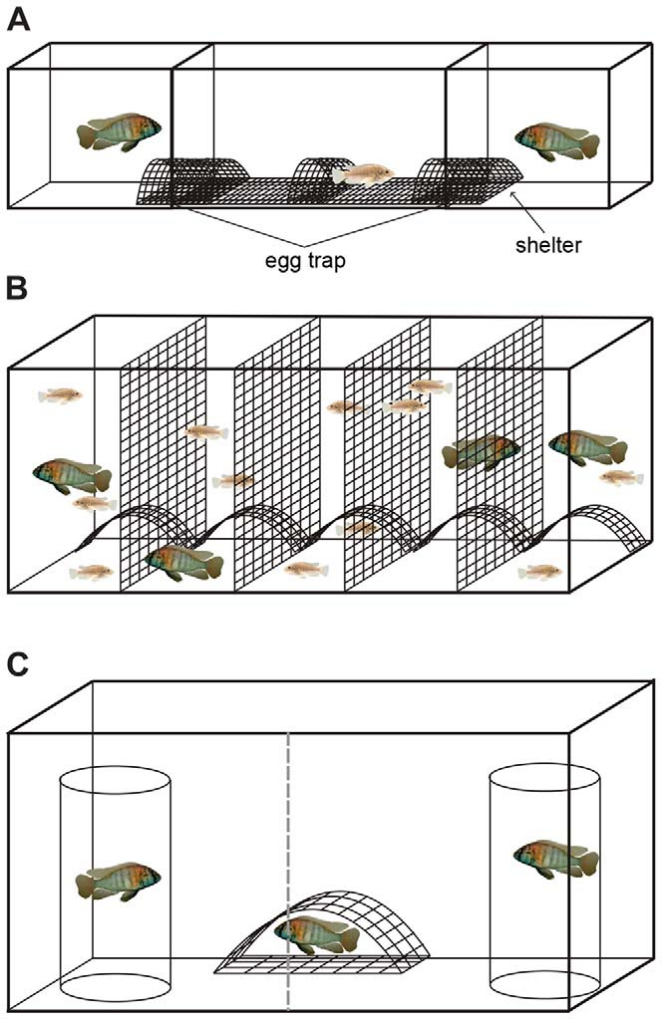
Experimental set-up (schematic view). (A) The two-way choice set-up showing the egg-traps and the shelters, which are permeable to eggs. (B) The four-way choice set-up (‘partial partition’ method) with semi-permeable grids, passable for females but not for males. (C) The set-up for the male aggression trials with stimulus males in plastic cylinders and the focal male hiding in the shelter.

**Figure 3:**
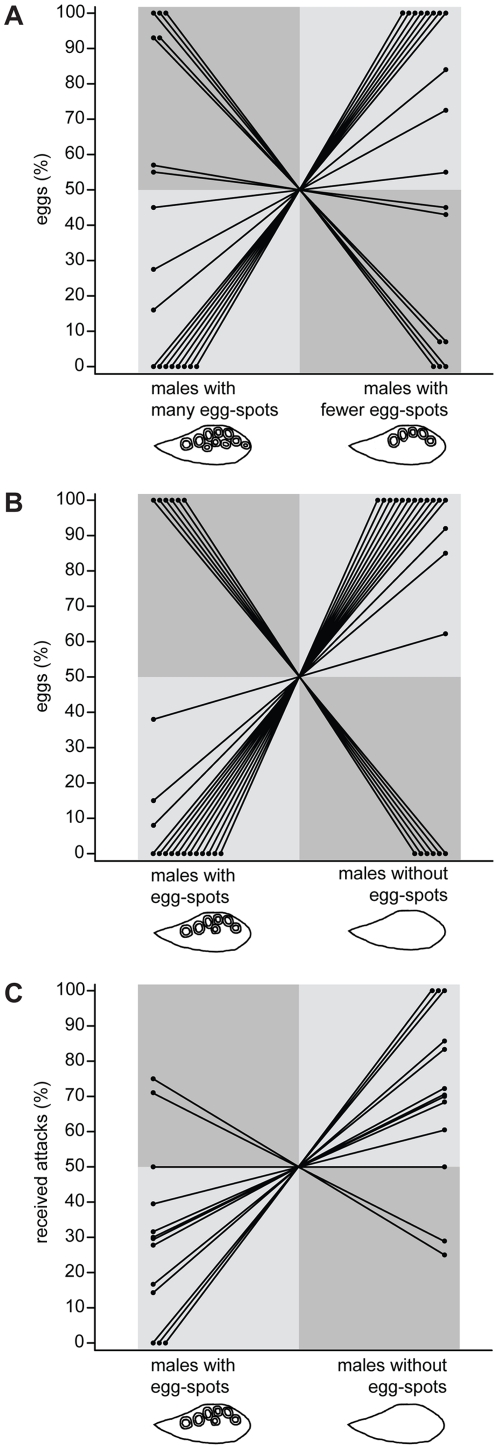
Percentage of eggs and attacks that males received in experiments 1 and 3. Dots connected through lines indicate male pairs used in experiments. Data points in dark shaded squares were coded as 1 (the male with more egg-spots received more than 50% of the eggs of a clutch laid by a female or more than 50% of attacks of the focal male), data points in light shaded squares were coded as 0 (the male with more egg-spots received 50% or less of the eggs of a clutch laid by a female or 50% or less of the attacks of the focal male) for further analyses with generalized linear mixed models (GLMMs). (A) In experiment 1.1, females showed no preference for males with many or fewer egg-spots. (B) In experiment 1.2, females tended to prefer males without egg-spots over males with egg-spots. (C) In experiment 3, focal males attacked stimulus males without egg-spots at a higher rate compared to stimulus males with egg-spots.

In our female two-way choice experiments two size-matched males were presented to a focal female in two outer tanks arranged on both sides of the central female tank (Figure 2A). In experiment 1.1, 7 females laid more eggs in front of the male with many egg-spots and 11 females laid more eggs in front of the male with fewer egg-spots. Males with many egg-spots were not more likely to receive more eggs from females than males with fewer egg-spots (GLMM, n = 18, z = −0.892, p = 0.373; Figure 3A). In experiment 1.2 only 6 females laid more eggs in front of the male with egg-spots and 15 females laid more eggs in front of the male without egg-spots. Thus, females tended to lay eggs preferentially close to males without egg-spots (GLMM, n = 21, z = −1.897, p = 0.058; Figure 3B). Most females laid the whole clutch in front of a single male but in 7 out of 18 trials in experiment 1.1 and in 3 out of 21 trials in experiment 1.2 the females laid eggs in front of both males.

### Experiment 2: female four-way choice

**Figure 4:**
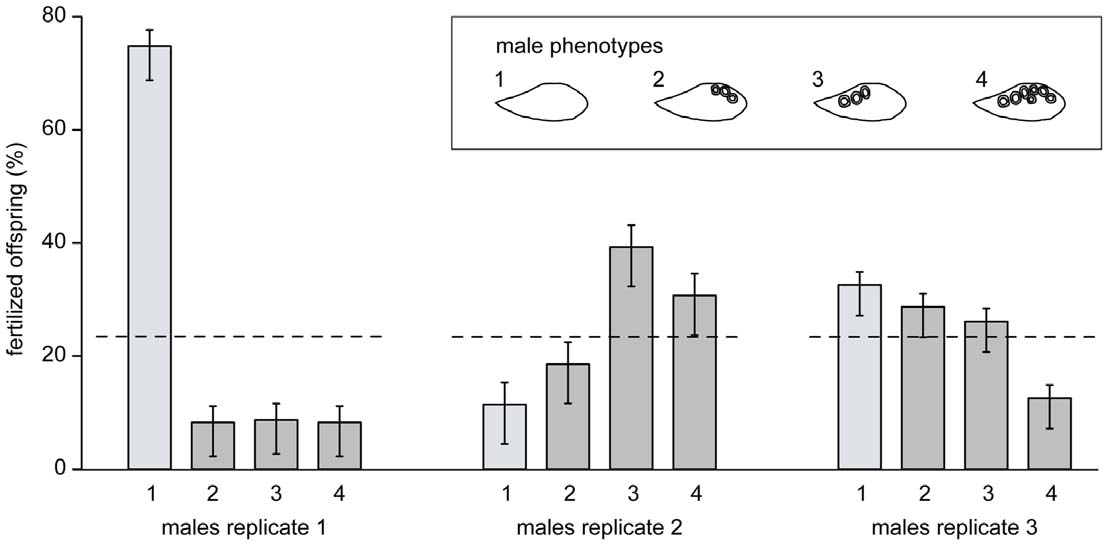
Results of experiment 2 (four-way choice). In replicate 1, females preferred the male without egg-spots (male phenotype 1) over the egg-spot bearing males (phenotype 2, 3 and 4), whereas a more balanced distribution of fertilized eggs was found in replicates 2 and 3. The dotted line indicates the 25% limit, indicating the expected distribution of fertilized offspring under random mating.

The four-way choice set-up consisted of a large tank with five equally sized compartments (‘partial partition’ design; Figure 2B). Once a female was mouthbrooding, we removed the eggs or larvae to determine the fertilization rate and to test for paternity using microsatellites. In the first replicate, the three males with egg-spots fertilized 75% or more of the offspring in 5 clutches and the male without egg-spots fertilized 25% or more of the offspring in 18 clutches. Therefore, females preferred the male without egg-spots (Binomial test, n = 23, p<0.001). In replicate 2 and 3, however, the males without egg-spots (fertilizing 25% or more of the offspring in 2 and 10 clutches, respectively) were not significantly preferred over males with egg-spots (fertilizing 75% or more of the offspring in 12 and 21 clutches, respectively; replicate 2: Binomial test, n = 14, p = 0.540; replicate 3: Binomial test, n = 31, p = 0.401; Figure 4). Due to the observation that within each replicate the three egg-spot bearing males weren't more attractive to females than males without egg-spots, we did not test for differences in the number of offspring sired by the three egg-spot bearing phenotypes. Fertilization rate was close to 100% in all cases, also in broods fathered by the male without egg-spots. A definitive female choice was found in 58 out of the 68 broods genotyped (93%), in which only one male sired the whole clutch. Multiple paternity was detected in 10 out of the 68 broods genotyped (7%). One out of these 10 broods was fathered by 3 different males, whereas 2 fathers were detected in the remaining 9 broods.

### Experiment 3: male aggression

To test the role of egg-spots in aggressive behavior, two size-matched males, one with unaltered and one with removed egg-spots, were placed in transparent cylinders and presented to a territorial male of similar size (Figure 2C). Out of 13 focal males, 10 showed a higher attack rate (bites, butts and quivers) towards stimulus males without egg-spots, 2 showed a higher attack rate towards stimulus males with egg-spots and 1 attacked both stimulus males at a same rate. Taken together, focal males attacked the stimulus males without egg-spots more often than the stimulus males with egg-spots (GLMM, n = 13, z = −2.218, p = 0.027; Figure 3C).

## Discussion

It is widely recognized that secondary sexual traits are targets of inter- or intrasexual selection, or both [4], [5], [16], [37], [38]. Sexual selection is often considered a central driving force in the evolution of the exceptionally colorful and species-rich haplochromine cichlid fishes endemic to rivers and lakes in East Africa [7], [9], [10]. One particular feature of haplochromines (at least of the derived ‘modern haplochromines’) is their possession of true egg-spots on the anal fins of males [12]. While previous work on the function of egg-spots solely focused on intersexual selection (female choice), we also tested, for the first time, for a putative role of egg-spots in intrasexual selection (male-male competition). Here, we focus on the haplochromine cichlid *Astatotilapia burtoni*, which is widely used in various kinds of experiments and whose genome has recently been sequenced (see e.g. [39], [40]).

The two-way choice experiments revealed that there is no female preference for many egg-spots in *A. burtoni* and that females even tended to prefer males without egg-spots. In these experiments, we used egg-traps to quantify if a focal female laid more eggs towards one of the naturally distinct males (experiment 1.1) or towards one of the males with an artificial difference in egg-spot number (experiment 1.2), as the actual egg-laying activity towards a male appears to be a better predictor of female preference than *e.g.* time-spent (see e.g. [41]). Our four-way choice experiment, with free contact in combination with paternity testing, corroborates that there is no preference for many egg-spots in *A. burtoni* females. While the different replicates did not reveal conclusive results with respect to a female preference for a certain number or arrangement of male egg-spots, this experiment clearly demonstrates that anal fin egg-spots are not required to attract females and to fertilize eggs.

The results from our female two- and four-way choice experiments contradict previous studies on the role of egg-spots in mate choice [23], [28] that did, however, not use the more accurate methods of egg-traps (two-way choice set-up) or paternity testing (four-way choice set-up). One biological explanation for the discrepancy between our results and previous ones might lie in the observation that egg-spot number correlates with the size of a male so that larger males generally display more egg-spots in the wild [42]. Females of *Astatotilapia elegans*, which prefer males with many egg-spots to males with fewer spots, also prefer larger males [43], whereas *A. burtoni* females prefer dominant yet smaller males that are more active during courtship [44].

The four-way choice experiment was also designed to detect possible differences in the number of fertilized eggs per clutch (fertilization rate) between the four males with distinct anal fin phenotypes. Obviously, there is no effect of number and arrangement of egg-spots on fertilization rate, as the number of fertilized eggs was 100% in nearly every clutch, which is in line with previous experiments in *A. elegans* [23]. Possibly, female haplochromines have fixed the snapping behavior towards the male anal fin after egg-laying and -uptake, so that egg-spots no longer act as visual triggers (*sensu* Wickler [20], [21]) – at least in those species tested so far. This is further corroborated by the relative position of the egg-spots on male anal fins, as they often occur at the terminal end rather than close to the genital opening. Moreover, intraspecific variation in egg-spot number (Figure 1B) would not be likely to prevail if egg-spots were necessary for a successful fertilization [34].

The genotyping of offspring and candidate parents in the four-way choice experiments allowed us to test for extra-pair fertilization in *A. burtoni*. Multiple paternity is rather common in haplochromines. A study on several lekking rock- and sand-dwelling species from Lake Malawi, for example, uncovered multiple paternity in almost every brood [45]. Here we show that multiple paternity also occurs in *A. burtoni* (at least under laboratory conditions), but that its frequency is low (10 out of 68 broods) compared to the natural situation in Lake Malawi cichlids.

Taken together, our female mate choice experiments demonstrate that egg-spots in *A. burtoni* do not serve as recognition pattern (at least on short distances) or attraction signal for females, and that they do not maximize fertilization rates. Still, egg-spots are present in most haplochromine species, indicating an important function additionally to the one in intersexual selection. It has previously been suggested that the honesty and genetic variance of a trait are actually easier maintained through male-male competition than through female choice [6]. Our new results indeed point towards a function of egg-spots in an intrasexual rather than an intersexual context: in a combat situation, males without egg-spots suffered from increased attack rates compared to males with intact egg-spots (Figure 3C), suggesting that egg-spots are an honest signal of male quality used in male-male competition (as carotenoid based ornaments [19], [22], egg-spots are likely to display health status or aggressiveness). It is important to note that this effect could only be observed when size-matched males were used; overall, the effect of body size, and possibly body weight, outbalances differences in egg-spot number [34]. Differences in egg-spot number might still have a profound effect in the wild, as males are likely to primarily encounter opponents of similar size. In nature, only 10–30% of the males are territorial [46] resulting in the situation that only the largest sized males are capable of establishing a territory and gain access to females. If those males fight, morphological or behavioral traits other than size – such as the colorful egg-spots – may become important. It has been shown in *A. burtoni* that the attack readiness of males was influenced by specific body patterns - e.g. the head pattern in form of a black bar increases and the orange patch on the cheek decreases number of bites from a competitor [47]. In our study, the stimulus males displayed all patterns known to indicate territorial status (e.g. territorial body coloration, black bar and orange patch) but the lack of egg-spots increased attacks of a competitor.

There are, in fact, other examples from cichlid fishes where coloration or pigmentation signals have an intimidating effect in male combats. Fights in the North American cichlid *Thorichthys meeki* (formerly known as *Cichlasoma meeki*), for example, became more violent when the ornament in form of an eye-spot had been removed [48]. In the Lake Victoria haplochromine genus *Pundamilia*, the advantage of more intensely colored males to win a fight (under white light) vanished under green light conditions masking the carotenoid-based red coloration [49]. Egg-spots in *A. burtoni* appear to exert a similarly intimidating effect on the competitor during threatening and fighting.

Females of several species are known to prefer males with increased carotenoid coloration, which indicates health status: in sticklebacks, for example, the red belly coloration functions as a threat signal in intrasexual competition but also in female mate choice [50]. Note that there are reverted examples, too: in red-collared widowbirds, females select against the male carotenoid display, which also has a dominance function in male-male competition [51], indicating that the display of male dominance and aggressiveness can also have intimidating effects on females. In general, however, such male displays are thought to aid keeping the attack levels in intrasexual competition within limits, as the aggression level and (health) status of rivals may be judged upon these displays [52], [53], [54]. With respect to the egg-spots of haplochromine cichlids it thus seems likely that the males with more (or more conspicuous) egg-spots are ranked as stronger competitors. These males are then the ones to establish a territory, which in turn gives them opportunity to mate, as social status and mating success are typically correlated in most cichlids [40], [55]. That way, females would choose males with more or brighter egg-spots indirectly by choosing males that can pay the competitive cost of gaining a high-quality territory.

Taken together, the involvement of egg-spots in female choice [23], [29] and in male aggression (our study) point towards multiple functions of egg-spots in haplochromine cichlids.

## Methods

### Study species

The cichlid species *Astatotilapia burtoni*, a maternal mouthbrooder, is a generalist living in the estuaries and affluent river systems of Lake Tanganyika, East Africa. As is typical for polygynous mating systems, the species shows sexual dimorphism: males are larger, more intensively colored and their egg-spots are much more pronounced and show, in contrast to female egg-spots, a hyaline circle, which is characteristic for ‘true egg-spots’ [56]. Phylogenetically, *A. burtoni* is member of a group of riverine haplochromines that are the sister group to the species flock of Lake Victoria region, and, together with the latter, the sister group to the Lake Malawi species assemblage [12], [57].

The female test animals were kept in a pure female tank (100×50×50 cm) and the males in mixed-sex stock tanks (100×50×50 cm), from which they were transferred into smaller individual tanks before testing. All tanks provided standardized conditions of constant water temperature of 26°C, pH 7, and a 12:12 h light:dark cycle. Flake food was fed twice a day and frozen artemia was given once a day. Our aquaria strain population, which was used in most of the experiments, originated from an inbred line. The wild caught specimens used in the four-way choice experiment were imported from the Kalambo region in Zambia in 2009.

All laboratory mate choice experiments were performed at the Zoological Institute of the University of Basel under the permission of the Cantonal Veterinary Office, Basel, Switzerland (permit numbers: 2356, 2403). Manipulations on anal fin egg-spots were performed under clove oil anesthesia (2–3 drops clove oil per liter water).

### Experiment 1: female two-way choice

A three-tank set up (see *e.g.* [29], [44], [58]) was used in order to test female preference based on visual cues alone. Two males were presented, in two outer tanks (40×25×25 cm), to the female, which was placed in a central tank (60×30×30 cm). All tanks were equipped with egg-traps in the form of a plastic grid (eggs would simply fall through the grid so that females could not take them up into their mouth). The outer male tanks contained one shelter (made from a plastic grid) each, whereas three shelters were placed in the central female tank (one on each males' side and one in the center; Figure 2A). Using this set-up, two female two-way choice experiments were conducted. In the first experiment (experiment 1.1) the males' egg-spots differed naturally; we tested males with many (n = 10; egg-spot number mean ± sd = 12.0±2.055, range 9–16) *versus* males with fewer egg-spots (n = 10; egg-spot number mean ± sd = 8.1±0.994, range 6–10). In the second experiment (experiment 1.2) artificial variation was created through experimental manipulations with dry ice to entirely remove all egg-spots (‘freeze-branding’ method; [23], [59]); we tested males with many (n = 11; egg-spot number mean ± sd = 11.7±2.005, range 9–15) *versus* males without egg-spots (n = 11). As a treatment control, both competitors of a trial were freeze-branded, with one male being treated directly on the egg-spots while the other one was treated below the egg-spots. Males of a male pair had similar territorial body coloration and were size-matched (using total length and weight), therefore they did not differ in size nor weight but in the number of anal fin egg-spots (experiment 1.1, Wilcoxon signed-rank test, n = 10: size V = 22, p = 1, weight V = 40, p = 0.221, egg-spot number V = 55, p = 0.005; experiment 1.2, Wilcoxon signed-rank test, n = 11: size V = 35.5, p = 0.859, weight V = 52, p = 0.102, egg-spot number V = 66, p = 0.004; Table S1A). Due to limitations in the number of similar-sized males we used eight male pairs in experiment 1.1 and ten pairs in experiment 1.2 twice. Two pairs in experiment 1.1 and one male pair in experiment 1.2 were tested only once.

To initiate an experimental run, males were given at least 24 hours (in experiment 1.1) or one week (in experiment 1.2 in which recovery from the freeze branding treatment was necessary) to acclimate to the outer tanks and to become territorial (as indicated by nuptial coloration and behavior). Then a gravid female (identifiable through swollen abdomen and enlarged papilla; experiment 1.1: n = 18; experiment 1.2: n = 21; Table S1A) was introduced in the central tank. Female and male behavior was recorded with a video camera (Sony handicam, DCR-HC90E PAL, 3.0 mega pixels). The female was left in the tank until she laid eggs or for a maximum of 7 days and the position of eggs in the egg trap (choice zone next to male 1 (12 cm) *versus* choice zone next to male 2 (12 cm)) was recorded. The percentage of eggs per clutch that a female laid in front of each male was calculated. Due to the fact that often all eggs were laid exclusively next to one male, causing zero inflation in the data, we coded the data as 1 if the male with egg-spots received more than 50% of the eggs and 0 if it received 50% or less of the eggs laid by the female compared to the male with fewer egg-spots (experiment 1.1) or without egg-spots (experiment 1.2). We applied generalized linear mixed models (GLMMs) with a logistic link function (LME4 package [60]), because the response variable was binary (male with egg-spots received more than 50% of the eggs versus male with egg-spots received 50% or less of the eggs). To account for the fact that some pairs of males were used twice, we included male pair as a random factor. We tested whether the probability that a male with egg-spots received more than 50% of eggs was significantly different from 0.5 (i.e. whether the intercept on the logit scale was different from 0). Statistical analyses were performed using the software R, version 2.14.0 [61].

### Experiment 2: female four-way choice

This round of experiments made use of the ‘partial partition method’ (see *e.g.* [16]). A large tank (150×50×50 cm) was divided into five equally sized compartments (30×50×50 cm), which were separated by a plastic grid. The chosen grid-size allowed the smaller females to migrate freely, whereas the larger males were restricted to a single compartment. In each compartment a halved flowerpot served as territory center and hiding place. The middle compartment served as a resting area for the females (Figure 2B).

Four different male phenotypes were produced by freeze branding: (1) no egg-spots; (2) half the amount of egg-spots (remaining at the end of the anal fin); (3) half the amount of egg-spots (remaining close to the genital opening); (4) all egg-spots present (freeze-branding was done at a different area of the fin as treatment control; Figure 4). The males were checked regularly and freeze-branding was repeated if egg-spot pigments reappeared. For this purpose the females were removed from the experimental tank as long as the males needed to recover from the treatment (between two and seven days).

Three replicates with four males each and constantly 12 to 20 females were conducted; males were matched by size and weight (Table S1B) and swapped regularly between compartments to avoid compartment effects. Once a female was mouthbrooding (Figure 1C) she was caught, measured, fin-clipped (for DNA extraction) and the eggs or larvae were removed from her buccal cavity. Fertilization rate was recorded by estimating the number of fertilized eggs or larvae *versus* unfertilized eggs. The fertilized eggs or larvae were incubated in an Erlenmeyer flask for one to six days until they were developed enough for DNA extraction. DNA of ten larvae of each of the total 68 clutches (replicate 1: 23 clutches, replicate 2: 14 clutches, replicate 3: 31 clutches), their corresponding mothers and the putative fathers were used for paternity testing with at least five available un-linked microsatellite markers using a multiplex approach (Qiagen multiplex kit). We used the following markers: Abur82 [62], HchiST68 [63], Osu22d [64], Ppun5, Ppun7, Ppun21 [65], Pzeb3 [66], UNH130 [67], and UNH989 [68]. The amplified DNA samples were genotyped on an Applied Biosystems (ABI) 3130*xl* genetic analyzer and sized in comparison to LIZ 500(-250) (ABI) internal size standard. Genotypes were determined manually using the Genemapper software (version 1.0, ABI). With this procedure the father of each fry could be determined and it became apparent if multiple paternity occurred. Similarly as in experiment 1, we coded the data as 1 if the three males with egg-spots sired 75% or more of the eggs and 0 if the male without egg-spots sired 25% or more of the eggs. The three replicates were analyzed separately using binomial tests with a probability of 0.75 to check if the three egg-spot bearing males had a benefit and therefore received a higher number of clutches.

### Experiment 3: male aggression

This set up consisted of a tank (60×30×30 cm) containing a shelter for the focal male (n = 13) and two transparent plastic cylinders (d = 9.5 cm, h = 27 cm), one for each stimulus male (Figure 2C). The focal male was introduced into the aquarium and allowed to acclimate for at least 24 hours. Then two size-matched males (one with intact egg-spots (n = 8; egg-spot number mean ± sd = 7.8±2.123; range 5–11) and the other without egg-spots (n = 8)) were each placed in cylinders to avoid injuries. Three stimulus male pairs were used once and five stimulus pairs were used twice in alternating positions. Males of a male pair had similar territorial body coloration and were size-matched (by size (total and standard length) and weight), therefore they did not differ in total length, standard length and weight, but they differed in the number of anal fin egg-spots as described above (Wilcoxon signed-rank test, n = 8: total length V = 22, p = 0.641, standard length V = 22.5, p = 0.575, weight V = 19, p = 0.945, egg-spot number V = 36, p = 0.014; Table S1C). We then recorded the behavior of the focal male towards the two intruders by counting the three aggressive behaviors bites, butts and quivers [34]. These measurements were analyzed for a period of ten-minutes (right after the first interaction of the focal male with a stimulus male) from a one-hour video (Sony handicam; see above). The total number of times an aggressive behavior of one of the three categories was performed was used as a total aggression rate for the analysis. Similar to the analysis of experiment 1, the data were coded as 1 if the male with egg-spots had a higher and 0 if it had the same or a smaller aggression rate as the male without egg-spots. Generalized linear mixed models (GLMMs) with binomial error distribution and male pair as a random factor was used to determine if the focal male reacted differently to males with or without egg-spots.

## Supporting Information

Table S1 **Measurements taken from test animals.** (A) Experiment 1.1 and 1.2. (B) Experiment 2. (C) Experiment 3.
(PDF)
