# Supplementary material for: The Function of Anal Fin Egg-Spots in the Cichlid Fish Astatotilapia burtoni
Source: PLoS One. 2012 Jan 5;7(1):e29878. doi: 10.1371/journal.pone.0029878 (PMC3252332; doi:10.1371/journal.pone.0029878)
Supplement: Table S1 — Measurements taken from test animals. (A) Experiment 1.1 and 1.2. (B) Experiment 2. (C) Experiment 3. (PDF) [file pone.0029878.s001.pdf]

A

experiment	type of test group	measurement	range of all individuals min - max	mean of all individuals ± sd	range of the difference between individuals of a pair min - max	mean of the difference between individuals of a pair ± sd
**experiment 1.1**	males (n_{pairs} = 10, n_{ind} = 20)	total length (mm)<br>weight (g)<br>egg-spots (num)	97 - 114<br>13.24 - 21.16<br>6 - 16	105.3 ± 4.22<br>16.83 ± 1.83<br>10.05 ± 2.54	0 - 3<br>0.24 - 2.98<br>2 - 8	1.6 ± 0.97<br>1.22 ± 0.91<br>3.90 ± 1.73
	females (n = 18)	total length (mm)<br>weight before testing (g)<br>weight after testing (g)<br>eggs laid (num)	61 - 83<br>3.73 - 9.08<br>3.41 - 8.04<br>7 - 123	7.12 ± 0.65<br>6.11 ± 1.62<br>5.38 ± 1.36<br>61.06 ± 33.89	-<br>-<br>-<br>-	-<br>-<br>-<br>-
**experiment 1.2**	males (n_{pairs} = 11, n_{ind} = 22)	total length (mm)<br>weight (g)<br>egg-spots artificial (num)<br>egg-spots original (num)	89.33 - 113.90<br>8.51 - 20.75<br>0 - 15<br>6 - 15	105.29 ± 7.81<br>16.20 ± 3.78<br>5.86 ± 6.16<br>10.55 ± 2.48	0.04 - 9.00<br>0.42 - 4.29<br>9 - 15<br>0 - 7	2.37 ± 2.73<br>1.45 ± 1.10<br>11.73 ± 2.00<br>2.36 ± 3.47
	females (n = 21)	total length (mm)<br>weight before testing (g)<br>weight after testing (g)<br>eggs laid (num)	62.23 - 82.19<br>3.89 - 9.08<br>3.23 - 7.71<br>30 - 133	75.00 ± 5.50<br>6.91 ± 1.34<br>6.08 ± 1.22<br>96.33 ± 31.00	-<br>-<br>-<br>-	-<br>-<br>-<br>-

B

experiment	type of test group	measurement	range of all individuals min - max	mean of all individuals ± sd	range of the difference between individuals of a group min - max
**experiment 2 - replica 1**	males (n_{group} = 1, n_{ind} = 4)	total length (mm)<br>standard length (mm)<br>weight (g)<br>egg-spots artificial (num)<br>egg-spots original (num)	96.24 - 99.58<br>75.60 - 79.16<br>11.94 - 14.32<br>0 - 11<br>6 - 11	97.38 ± 1.51<br>78.23 ± 1.75<br>12.96 ± 1.21<br>5.25 ± 4.57<br>8 ± 2.16	0.38 - 3.34<br>0.05 - 3.56<br>0.00 - 2.38<br>2 - 11<br>1 - 5
	females (n = 23)	total length (mm)<br>standard length (mm)<br>weight after testing (g)<br>eggs laid (num)	4.60 - 6.34<br>NA<br>1.50 - 3.32<br>15 - 79	5.43 ± 0.48<br>NA<br>2.30 ± 0.52<br>44.35 ± 15.92	-<br>-<br>-<br>-
**experiment 2 - replica 2**	males (n_{group} = 1, n_{ind} = 4)	total length (mm)<br>standard length (mm)<br>weight (g)<br>egg-spots artificial (num)<br>egg-spots original (num)	85.16 - 86.73<br>67.10 - 68.72<br>8.20 - 8.55<br>0 - 7<br>6 - 11	85.99 ± 0.65<br>68.19 ± 0.74<br>8.3 ± 0.17<br>3.75 ± 2.87<br>7.5 ± 2.38	0.28 - 1.57<br>0.07 - 1.62<br>0.01 - 0.35<br>3 - 7<br>0 - 5
	females (n = 14)	total length (mm)<br>standard length (mm)<br>weight after testing (g)<br>eggs laid (num)	47.87 - 64.51<br>38.97 - 52.68<br>1.08 - 3.90<br>18 - 59	53.36 ± 5.86<br>43.01 ± 4.86<br>2.08 ± 0.85<br>32.21 ± 13.40	-<br>-<br>-<br>-
**experiment 2 - replica 3**	males (n_{group} = 1, n_{ind} = 4)	total length (mm)<br>standard length (mm)<br>weight (g)<br>egg-spots artificial (num)<br>egg-spots original (num)	97.39 - 99.91<br>77.38 - 79.86<br>12.00 - 14.54<br>0 - 5<br>5 - 6	98.46 ± 1.16<br>79.12 ± 1.17<br>13.46 ± 1.18<br>2.75 ± 2.06<br>5.25 ± 0.50	0.28 - 2.52<br>0.12 - 2.48<br>0.26 - 2.54<br>2 - 5<br>0 - 1
	females (n = 31)	total length (mm)<br>standard length (mm)<br>weight after testing (g)<br>eggs laid (num)	46.40 - 61.35<br>37.09 - 49.49<br>1.24 - 3.02<br>14 - 64	53.98 ± 3.81<br>43.37 ± 3.52<br>2.14 ± 0.43<br>35.39 ± 13.88	-<br>-<br>-<br>-

C

experiment	type of test group	measurement	range of all individuals min - max	mean of all individuals ± sd	range of the difference between individuals of a pair min - max	mean of the difference between individuals of a pair ± sd
**experiment 3**	stimulus males (n_{pairs} = 8, n_{ind} = 16)	total length (mm)<br>standard length (mm)<br>weight (g)<br>egg-spots artificial (num)<br>egg-spots original (num)	85.77 - 103.43<br>66.60 - 80.41<br>7.92 - 15.24<br>0 - 11<br>5 - 11	94.22 ± 5.93<br>73.13 ± 4.90<br>11.33 ± 2.46<br>3.88 ± 4.26<br>7.71 ± 1.90	0.01 - 2.18<br>0.01 - 2.07<br>0.21 - 1.18<br>5 - 11<br>1 - 4	1.02 ± 0.97<br>1.43 ± 0.80<br>0.82 ± 0.48<br>7.75 ± 2.12<br>2.17 ± 1.17
	focal males (n = 13)	total length (mm)<br>standard length (mm)<br>weight (g)<br>egg-spots (num)	86.02 - 108.85<br>66.78 - 87.08<br>8.19 - 18.38<br>6 - 11	93.90 ± 7.51<br>73.14 ± 6.67<br>10.97 ± 2.88<br>8.31 ± 1.49	-<br>-<br>-<br>-	-<br>-<br>-<br>-
